# The “5–4-3” model for weight management in psychiatric inpatients: a single-arm pre-post evaluation

**DOI:** 10.3389/fpubh.2026.1807147

**Published:** 2026-06-18

**Authors:** Huixian Xie, Huiping Yuan, Yiping Tang, Lingmei Lu, Jucai Chu

**Affiliations:** 1 Department of Nursing, Taizhou Second People's Hospital, Taizhou, China; 2 Department of Psychiatry, Taizhou Second People's Hospital, Taizhou, China; 3 Department of Psychosomatic Medicine, Taizhou Second People's Hospital, Taizhou, China; 4 School of Pharmaceutical Business & School of Digital Medical Technology, Zhejiang Pharmaceutical University, Ningbo, China

**Keywords:** closed-loop management, inpatients, multidisciplinary team, psychiatry, weight management

## Abstract

**Background:**

Comprehensive weight management remains an understudied yet critical component of inpatient psychiatric care, particularly for individuals presenting with overweight or obesity. To address this gap, the present study developed and evaluated the feasibility and preliminary effectiveness of a structured intervention termed the “Five-Discipline, Four-Phase, Three-Support” closed-loop weight management model (5–4-3 Model). In this single-arm pre-post study conducted among psychiatric inpatients at Taizhou Second People’s Hospital. We evaluated the model across multiple domains, including weight reduction and maintenance, patient-centered health outcomes, and healthcare team competency.

**Methods:**

A single-arm pre-post study was conducted from November 2024 to April 2025. Psychiatric inpatients with a body mass index (BMI) ≥ 24 kg/m^2^ were enrolled. The core intervention was the implementation of the 5–4-3 Model, a multidisciplinary closed-loop management system led by teams from psychiatry, nursing, clinical nutrition, rehabilitation medicine, and clinical pharmacy. The intervention comprised inpatient care (with a length of stay ranging from 1 to 6 months) and a 3-month post-discharge follow-up. Weight at 3-month follow-up was primarily obtained via patient self-report, introducing potential reporting bias. Outcome measures included changes in body weight, BMI, and metabolic indices (extracted from hospital records), patients’ weight management knowledge (assessed via a self-administered questionnaire), and staff awareness of weight management protocols and completeness of weight-related data recording. Data were analyzed using paired-sample t-tests.

**Results:**

All 139 enrolled patients completed the 3-month follow-up assessment. Significant reductions were observed in body weight (mean change from admission to 3-month follow-up: −2.95 kg; 95% CI -3.51 to −2.38; *p* < 0.001) and BMI (mean change: −1.10 kg/m^2^; 95% CI -1.28 to −0.92; *p* < 0.001). Patients weight management knowledge scores increased from 27.82 to 53.45% (*p* < 0.001). Post-intervention improvements were also noted in staff awareness of weight management protocols and the completeness of weight-related documentation.

**Conclusion:**

The 5–4-3 Model suggested preliminary feasibility and was associated with short-term improvements in weight, metabolic markers, and knowledge. However, causal attribution is limited by the uncontrolled single-arm design, and these findings should be considered preliminary. Controlled trials are needed to confirm efficacy.

## 1 Introduction

Overweight, obesity, and their associated metabolic diseases represent a major global public health challenge (1–3). In China, the rising prevalence of these conditions parallels ongoing socio-economic development and lifestyle changes. In response, China’s National Health Commission launched the “Weight Management Year” initiative and promulgated the “Weight Management Guiding Principles” in 2024 (4). These policies mandate the integration of comprehensive weight management services into chronic disease prevention frameworks across healthcare institutions.

The situation among psychiatric inpatients is particularly critical. Psychotropic drug-related weight gain (PDWG) is a common and serious adverse effect of many antipsychotic and antidepressant medications (5, 6). This iatrogenic effect reduces treatment adherence and satisfaction and increases the risk of developing metabolic syndrome, type 2 diabetes, cardiovascular diseases, and other potentially life-threatening conditions (7, 8). Notably, our study enrolled patients with pre-existing overweight or obesity (BMI ≥ 24 kg/m^2^) at admission, reflecting a high-risk population that includes but is not limited to PDWG. The prevalence of overweight and obesity in this population far exceeds that of the general population, contributing to a substantial reduction in average life expectancy. Therefore, effective weight management is a crucial component of holistic care for patients with severe mental illness.

Despite clear policy directives and clinical necessity, significant implementation barriers exist. Macro-systemically, standardized management pathways tailored to psychiatry are lacking. Although structured metabolic management models have been reported in psychiatric outpatient settings (9), inpatient ward models with continuity of care through discharge remain limited. Meso-organizationally, psychiatric hospitals face challenges such as fragmented interdisciplinary collaboration, limited staff expertise, and inadequate assessment tools. Micro-clinically, weight management is often not embedded into core treatment workflows, resulting in fragmented interventions. Furthermore, patient non-adherence due to psychiatric symptoms and difficulties in outcome tracking are common (10). This multi-level “systemic gap” undermines consistent and effective care delivery.

**Figure 1:**
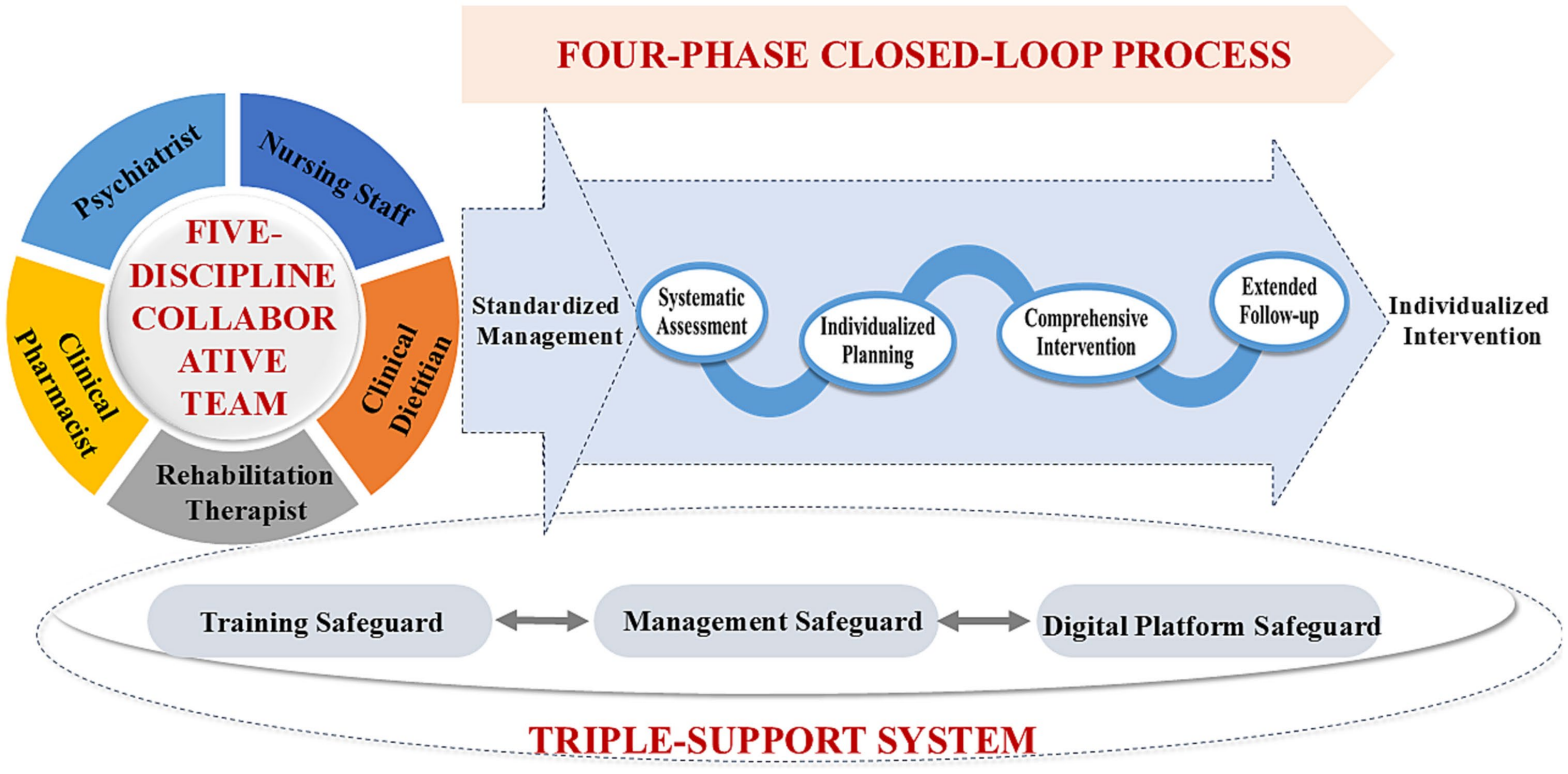
The 5–4-3 Model. This schematic illustrates the three core components of the 5–4-3 model and their interrelationships. The five-discipline collaborative team (on the left) is responsible for executing the process. The four-phase closed-loop process (in the center) represents the standardized clinical pathway from admission assessment to post-discharge follow-up. The triple-support system (at the bottom) provides foundational support for implementation. Solid arrows within the four-phase process indicate the direction of workflow, while double-headed arrows within the support system highlight synergistic relationships among the safeguards.

Consequently, there is an urgent need to develop and evaluate a standardized, integrated weight management model suitable for psychiatric inpatient settings. Based on a practical initiative at Taizhou Second People’s Hospital, this study aimed to construct and preliminarily assess a closed-loop management model, the Integrated “Five-Discipline, Four-Phase, Three-Support” Model (hereinafter referred to as the 5–4-3 Model, Figure 1). The research question was framed using a standardized PICO(T) framework for methodological clarity: the target population comprised psychiatric inpatients with overweight or obesity; the intervention was the 5–4-3 multidisciplinary closed-loop weight management model; comparisons were made via pre-post within-group self-comparison; outcomes included changes in body weight, BMI, metabolic indicators, patient health knowledge, and healthcare team competency; and the study timeframe encompassed inpatient intervention plus a 3-month post-discharge follow-up. The specific objectives were: (1) to detail the model’s components and operational mechanisms; (2) to analyze its impact on management process efficiency (3) to preliminarily evaluate its effects on patient metabolic indicators and healthcare team performance. This study provides an actionable framework to bridge the policy-practice gap and improve metabolic health outcomes in psychiatric care.

## 2 Methods

### 2.1 Study design

A pre- and post-intervention study was conducted to examine the feasibility and preliminary effectiveness of the newly developed 5–4-3 model, an integrated closed-loop weight management model for psychiatric inpatients. This early-phase pragmatic implementation study was guided by the Plan-Do-Study-Act (PDSA) cycle, which is widely recommended for practice-based improvement initiatives (11) and has been successfully applied in recent implementation research in mental health settings to introduce complex interventions, such as suicide prevention protocols in primary care (12).

In this study, a single-group pre-post test design was adopted, which was selected to inform the development of future large-scale randomized controlled trials (RCTs) rather than to establish definitive causal inference. The 5–4-3 model was implemented and prospectively observed over a 6-month period in the psychiatric inpatient wards of Taizhou Second People’s Hospital, with key parameters related to subsequent RCT design being collected and estimated.

### 2.2 Study setting and participants

The study was conducted in the psychiatric inpatient department of Taizhou Second People’s Hospital. Participants included patients admitted between November 2024 and April 2025 who met the following criteria:

Inclusion criteria (1): aged 17–79 years (2); primary diagnosis of schizophrenia or bipolar disorder (3); body mass index (BMI) ≥ 24 kg/m^2^ (defined as overweight or obese) (4); expected hospital stay ≥ 4 weeks; and (5) informed consent provided by the patient or legal guardian.

Exclusion criteria (1): severe comorbid physical illness (e.g., uncontrolled heart failure, advanced cancer) (2); pregnancy or lactation; or (3) inability to cooperate with baseline assessments due to severe agitation, stupor, or similar clinical conditions.

### 2.3 Intervention: the 5–4-3 model

The core intervention was the construction and implementation of a systematic weight management pathway based on the 5–4-3 Model, comprising three core components: a collaborative team structure, a closed-loop clinical process, and an enabling support system.

#### 2.3.1 Five-discipline collaborative team

We assembled a fixed multidisciplinary team, including specialists from psychiatry, nursing, clinical nutrition, rehabilitation medicine and clinical pharmacy, with clearly defined roles, mirroring previously reported models in psychiatric settings (13). Psychiatrists served as team leaders, responsible for diagnosing/treating mental illnesses, evaluating and adjusting antipsychotic medication regimens, and prioritizing agents with minimal metabolic impact in alignment with integrated metabolic management principles (8). Nursing Staff were tasked with daily monitoring and recording of patient weight and vital signs, as well as delivering basic health education on weight management. Clinical dietitians conducted nutritional risk screening using the Nutritional Risk Screening 2002 (NRS-2002) scale and developed individualized meal plans tailored to patients’ metabolic status and dietary preferences. Rehabilitation therapists assessed patients’ motor function and exercise willingness to formulate stratified, safe exercise prescriptions. Clinical pharmacists reviewed medication orders, evaluated the potential metabolic effects of prescribed drugs, and provided pharmaceutical recommendations to optimize treatment plans.

#### 2.3.2 Four-phase closed-loop management

The clinical workflow was structured into a standardized four-phase closed-loop process to ensure continuous care from hospital admission to community reintegration (14). First, a systematic assessment was completed within 72 h of admission. This phase supplemented routine psychiatric evaluation with a “three-dimensional metabolic risk assessment,” including nutritional screening via the NRS-2002, fasting blood tests for glucose and lipid profiles, and evaluation of patients’ activity willingness and baseline physical function to enable early identification of metabolic risks. Within one week after the assessment, the individualized planning phase was initiated, during which the multidisciplinary team collaboratively developed a written weight management plan for each patient. This plan established staged weight-loss goals and integrated coordinated strategies including medication adjustment, personalized nutrition guidance, graded exercise interventions, and behavioral support. Nutritional plans were individualized with caloric targets typically set at a 300–500 kcal/day deficit. Exercise prescriptions consisted of 30 min of moderate-intensity aerobic activity (e.g., brisk walking) at least 5 days per week, with progression based on patient tolerance. Throughout hospitalization, comprehensive interventions were embedded into daily care, such as a dedicated health education corner, routine reviews of metabolic indicators during ward rounds, biweekly thematic education sessions, and an exercise logbook to monitor adherence. Health education was delivered via biweekly 45-min group sessions covering dietary management, physical activity, and medication adherence. Finally, following discharge, an extended follow-up phase was implemented via a structured “three-tier follow-up mechanism.” Case management nurses, dietitians, and attending physicians conducted hierarchical follow-ups through telephone calls, WeChat communications, and scheduled outpatient visits over a 3- to 6-month period. To minimize reporting bias, patients were instructed to measure body weight using standardized home weighing scales and record the exact date and time of measurements during follow-up, although objective verification of these records was not performed. Body weight data at the 3-month post-discharge follow-up were mainly patient-reported, which may introduce potential reporting bias; this limitation is further discussed in the Discussion section.

#### 2.3.3 Triple-safeguard mechanisms

To ensure model fidelity, sustainability, and quality, three parallel support mechanisms were implemented. A training safeguard involved a “layered training program” (15), where core team members received advanced thematic instruction, all ward staff participated in mandatory monthly foundational training, and a structured “mentorship” system was used for new staff, with competency assessment required for model delivery. The management safeguard integrated key weight management performance indicators, such as screening completion rate and consultation response time, into both departmental and individual performance evaluations. This was further reinforced by a dedicated quality control team, which conducted regular random audits of process data to ensure compliance and accuracy. Concurrently, a digital platform safeguard was implemented via an upgrade to the hospital’s electronic medical record system. This upgrade introduced a dedicated “Weight Management Module,” which enabled the centralized entry, automatic calculation, and visual presentation of patient metabolic data. These features were designed to reduce manual error and enhance operational efficiency.

### 2.4 Assessment indicators

The evaluation focused on both the effectiveness of the management process and patient/staff outcomes.

Process Indicators: These included the average response time for multidisciplinary team consultations and the coverage rate of weight management interventions for patients with obesity. Due to incomplete system records and data standardization limitations, reliable process indicator data amenable to statistical analysis could not be obtained; therefore, these indicators are not reported in the results.

Patient Outcome Indicators: The primary indicators were changes in body weight and BMI during hospitalization and at 3 months post-discharge. Secondary indicators included changes in metabolic markers (fasting blood glucose and triglycerides) and the rate of patient knowledge awareness regarding weight management, which was assessed using a self-designed objective knowledge questionnaire.

Healthcare Staff Outcome Indicators: The primary indicators were changes in staff awareness and acceptance of the structured weight management protocol, evaluated using a self-designed objective knowledge questionnaire administered before and after model implementation to all 31 core team members. Additionally, the completeness rate of weight-related data entries in the electronic medical records was audited.

Prior to formal data collection, items were developed based on the 2024 Chinese Weight Management Guidelines and clinical metabolic management practice for psychiatric patients. A pilot test was conducted among 30 patients to refine wording and comprehension. The questionnaires used objective single-choice and true-false items with clear correct answers, using a standard scoring method. Three independent specialists in psychiatry, psychiatric nursing, and metabolic management reviewed all items and reached unanimous agreement to confirm content validity. Test–retest reliability was not formally assessed due to the pragmatic nature of this feasibility study, which is a limitation.

### 2.5 Data collection and statistical analyses

Data were collected from the hospital information system, self-administered questionnaires, and standardized assessment tools. Categorical data are reported as numbers and percentages. Continuous data are presented as mean ± standard deviation. The normality of difference scores was examined using the Shapiro - Wilk test; given the large sample size (*n* = 139), the paired t-test is considered robust to minor deviations from normality (16). Changes in body weight and BMI from admission to discharge and from admission to the 3-month post-discharge follow-up were assessed using paired-sample t-tests. For primary and secondary outcomes, effect sizes (Cohen’s *d*) were calculated using the formula *d* = *t* / √n for paired designs, where *t* is the t-statistic and *n* is the number of pairs. Effect sizes were interpreted as small (*d* = 0.2), medium (*d* = 0.5), or large (*d* = 0.8) according to Cohen’s guidelines.

### 2.6 Sample size

A formal sample size calculation is not required for a feasibility study. However, to get initial estimates of effects, at least 90 participants were needed in the study. This was calculated in relation to the chosen power (80%) and significance level (5%) two- tailed and the desired small effect size (0.3). After accounting for a 20% potential attrition rate, we aimed to enroll at least 113 participants.

**Figure 2:**
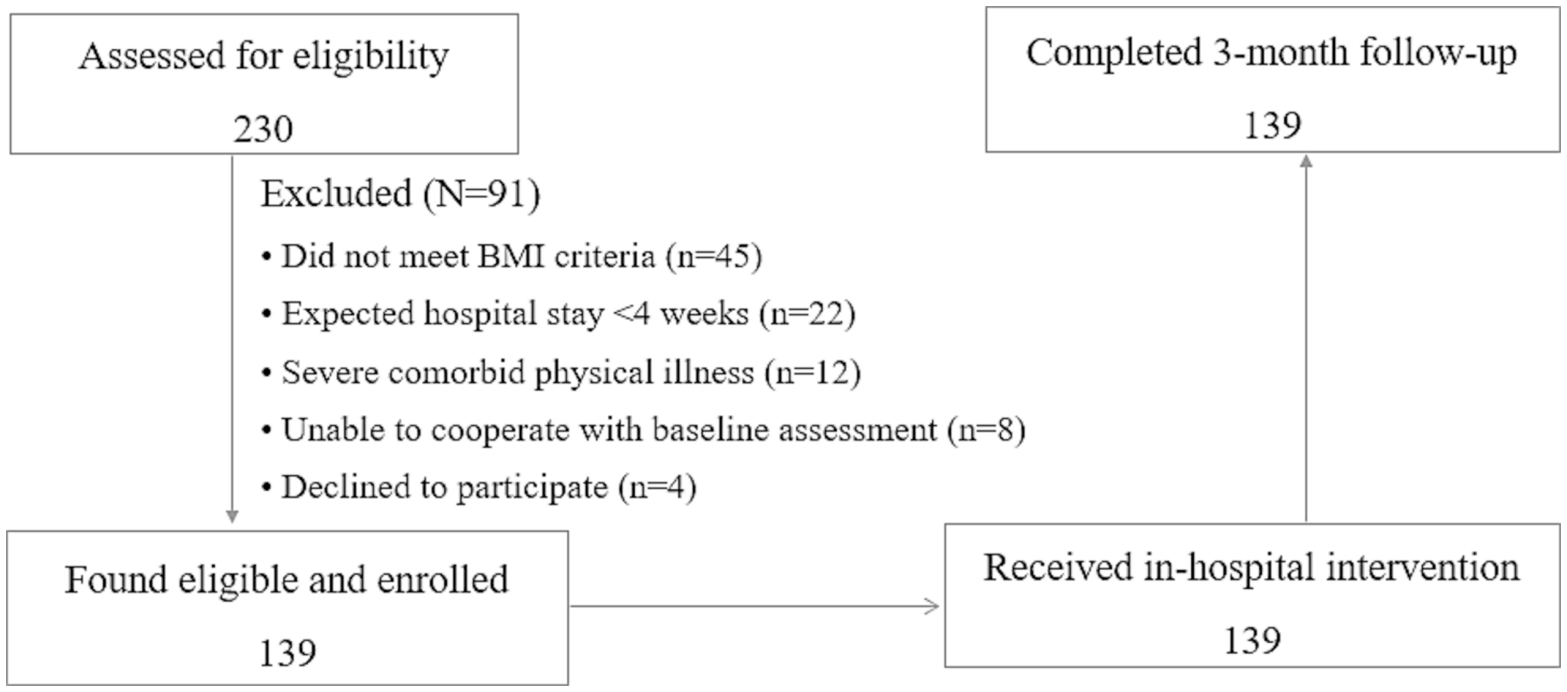
Participant flow diagram. A total of 230 patients were screened. After exclusions (*n* = 91) for reasons shown, 139 eligible patients were enrolled. All 139 completed the inpatient intervention and the 3-month post-discharge follow-up.

The participant flow diagram is depicted in Figure 2. In total, 230 patients were screened, and 139 eligible patients were enrolled. All 139 enrolled patients had complete outcome data at discharge and completed the 3-month post-discharge follow-up assessment. In addition, changes in the implementation capacity of the fixed multidisciplinary care team (*n* = 31) involved in delivering the intervention were evaluated.

## 3 Results

Table 1 reports the baseline characteristics of participants. Table 2 reports significant reductions from admission to discharge in body weight [MD = −1.33 kg, 95% CI (−1.65, −1.01), *p* < 0.001], BMI [MD = −0.50 kg/m^2^, 95% CI (−0.62, −0.38), *p* < 0.001], and fasting blood glucose [MD = −0.46 mmol/L, 95% CI (−0.62, −0.30), *p* < 0.001]. At the 3-month post-discharge follow-up, further improvements were observed from discharge to follow-up in body weight [MD = −1.62 kg, 95% CI (−2.04, −1.19), *p* < 0.001] and BMI [MD = −0.60 kg/m^2^, 95% CI (−0.76, −0.45), *p* < 0.001] and from admission to follow- up in body weight [MD = −2.95 kg, 95% CI (−3.51, −2.38), *p* < 0.001] and BMI [MD = −1.11 kg/m^2^, 95% CI (−1.31, −0.89), *p* < 0.001]. In contrast, the reduction in triglyceride levels observed at discharge did not reach statistical significance [MD = −0.09 mmol/L, 95% CI (−0.20, 0.02), *p* = 0.117]. Effect sizes. For weight reduction from admission to discharge, Cohen’s *d* was −0.689 [95% CI (−0.873, −0.503)], indicating a moderate-to-large effect. From admission to 3-month follow-up, the effect size increased to −0.876 [95% CI (−1.070, −0.679)], representing a large effect. Similarly, BMI reduction showed a moderate-to-large effect at discharge [*d* = −0.710, 95% CI (−0.889, −0.523)] and a large effect at follow-up [*d* = −0.889, 95% CI (−1.084, −0.691)]. Fasting glucose reduction from admission to discharge yielded a moderate effect [*d* = −0.497, 95% CI (−0.677, −0.315)], while triglyceride reduction was negligible [*d* = −0.135, 95% CI (−0.303, −0.034)], consistent with the non-significant *p*-value.

**Table 1:** Baseline characteristics of participants.

Characteristics	*N* (%) 139 (100%)
Age (years)	46.6 (12.5)*
Sex	
Male	96 (69.1%)
Female	43 (30.9%)
Antipsychotic regimen at admission	
Monotherapy	88 (63.3%)
Polytherapy (≥2 antipsychotics)	51 (36.7%)
Primary diagnosis	
Schizophrenia	112 (80.6%)
Bipolar disorder	27 (19.4%)
Metabolic risk category (highest risk agent if polytherapy)^a^	
High risk (olanzapine, clozapine)	102 (73.4%)
Moderate risk (risperidone, quetiapine, paliperidone)	31 (22.3%)
Low risk (aripiprazole, lurasidone)	3 (2.2%)
Not on antipsychotics (only mood stabilizers)	3 (2.2%)
Weight (kg)	74.95 (9.29)*
BMI (kg/m^2^)	27.79 (2.66)*
BMI category	
Overweight (24.0–27.9)	89 (64.0%)
Obesity (≥28.0)	50 (36.0%)

a. Metabolic risk categories for antipsychotics are based on the Chinese guideline for the diagnosis and treatment of schizophrenia (2025 version).

*Mean ± SD.

**Table 2:** Changes in Weight/BMI and metabolic indicators during hospitalization and 3 months post-discharge.

Index	Admission	Discharge	Difference between discharge and admission (95% CI)	*P*-value	3 Months post-discharge	Difference between 3-month post-discharge and Admission (95% CI)	Difference between 3-month post-discharge and discharge (95% CI)	*P*-value
Weight (kg)	74.95 (9.29)*	73.61 (9.50)*	−1.33 (−1.65 to −1.01)	<0.001	72.00 (9.91*)	−2.95 (−3.51 to −2.38)	−1.62 (−2.04 to −1.19)	<0.001
BMI (kg/m^2^)	27.79 (2.66)*	27.29 (2.71)*	−0.50 (−0.62 to −0.38)	<0.001	26.69 (2.88)*	−1.11(−1.31 to −0.89)	−0.60 (−0.76 to −0.45)	<0.001
Fasting glucose (mmol/L)	5.52 (1.43)*	5.06 (0.96)*	−0.46 (−0.62 to −0.30)	<0.001	-	-	-	
Triglycerides (mmol/L)	1.75 (0.78)*	1.67 (0.65)*	−0.09 (−0.20 to 0.02)	0.117	-	-	-	

*Mean ± SD; *n* = 139.

Simultaneously, patients demonstrated substantial improvements in their awareness of weight management following the regular health education provided. The mean score on the health knowledge questionnaire rose from 27.82 ± 19.1% at baseline to 53.45 ± 29.2% after the intervention, a difference that was statistically significant (*t* = 8.234, *p* < 0.001). Correspondingly, healthcare staff surveyed demonstrated a marked increase in their overall awareness of the weight management protocol. The mean score on the staff awareness questionnaire increased from 77.19 ± 12.71 at baseline to 99.03 ± 2.70 after the training, representing a mean improvement of 23.83 points [95% CI (19.00, 28.67); t (30) = 10.06, *p* < 0.001].

## 4 Discussion

The observed improvements in weight-related outcomes and health behaviors may be interpreted within the framework of Social Cognitive Theory (SCT), a well-established middle-range theory of health behavior change. SCT highlights the roles of self-efficacy, self-regulation, affective states, structured guidance, multidisciplinary support, and environmental reinforcement, all of which were embedded within the 5–4-3 closed-loop model to facilitate sustained behavioral modification. Recent research shows that early changes in self-regulation and mood during obesity interventions predict long-term improvements in self-efficacy and weight loss maintenance (17), supporting the SCT-based mechanisms underpinning our intervention, particularly the interplay between self-regulation, affect, and self-efficacy.

The implementation of the 5–4-3 Model demonstrated favorable clinical feasibility within a psychiatric inpatient setting. The model was associated with measurable improvements in patient outcomes, including weight loss during hospitalization that was maintained at the 3-month follow-up, as well as positive trends in key metabolic parameters such as fasting blood glucose. These findings support the feasibility of integrating weight management into routine psychiatric care. The model’s effectiveness is rooted in its systematic integration of three core components: a structured care process, multidisciplinary collaboration, and an enabling support system. By establishing a fixed team and a standardized closed-loop pathway from screening to follow-up, the intervention directly addressed common systemic challenges in psychiatric weight management, such as fragmented care and unclear accountability. This structured, procedural approach aligns with established chronic disease management frameworks that emphasize multidisciplinary coordination. The clear definition of roles and regular team interactions facilitated coherent action across specialties, translating policy intent into sustained clinical practice and ensuring consistent delivery of interventions.

A multidimensional risk assessment was launched at admission, and monitoring and education were incorporated into daily routines. These practices were central to embedding weight management as an integral part of holistic treatment. The intervention targeted physiological and behavioral mechanisms via individualized nutrition and exercise plans, complemented by health education that significantly improved patients’ health knowledge. This focus on health literacy is particularly pertinent for psychiatric patients, who may face motivational or cognitive barriers to self-management, which have been associated with metabolic dysregulation in mood disorders (18). The lack of significant change in triglyceride level may be partially explained by the high proportion of patients on lipid-lowering agents at baseline, although this was not systematically recorded; therefore, this should be interpreted as a hypothesis rather than a definitive finding. This is consistent with findings from other studies involving medicated patients.

The significant improvement in team execution capability confirms the importance of a supportive ecosystem for changing clinical practice (19). Healthcare staff’s mean awareness score of the protocol showed a substantial increase after the intervention (mean improvement of 23.83 points, *p* < 0.001), effectively aligning team cognition and reducing knowledge gaps. At the same time, the management safeguard provided sustained motivation through integrated performance indicators, while the digital platform safeguard optimized workflow efficiency. The model relied on threefold support: layered training, performance evaluation, and digital tools. These components worked synergistically. Their synergy helped healthcare staff move from initial awareness to routinized practice. It also secured the model’s adoption and long-term sustainability. Our inpatient closed-loop approach complements existing outpatient metabolic management experiences by providing continuity of care through the discharge transition (9). Meanwhile, community-based lifestyle interventions have also shown promise for improving metabolic health in this population (20).

This study has a number of strengths and weaknesses. First, as a single-group pre-post pragmatic study without a parallel control group, it cannot fully exclude the potential influence of temporal trends, concurrent hospital routine changes, or natural adjustments to clinical care pathways during the study period. The observed weight and metabolic improvements may also be partly related to the hospitalization environment, regular care, or other non-specific factors rather than the independent effect of the 5–4-3 Model. Therefore, the results only support preliminary feasibility and potential effectiveness rather than definitive efficacy. Future research should employ randomized controlled trials to provide higher-level evidence. Second, medication-related confounding remains a critical issue. Weight changes among psychiatric inpatients are closely linked to antipsychotic type, dosage, and metabolic risk. Although we classified metabolic risk at admission using a hierarchical approach (highest-risk agent for polytherapy), we did not systematically track dosage changes or medication adjustments during hospitalization. Dose reductions or switches to lower-risk agents could independently contribute to weight loss, whereas dose increases might counteract intervention effects. Without stratification by metabolic risk or adjustment for time-varying medication variables, we cannot fully disentangle the independent effect of the 5–4-3 Model from medication-related changes. Future studies should collect longitudinal medication records and perform stratified or sensitivity analyses to account for this confounding. Third, this was a single-center study with a highly motivated clinical team, so the generalizability of the model’s effectiveness to resource-limited primary care settings with varying implementation capacities remains to be verified. Finally, partial post-discharge follow-up data, particularly home-measured body weight, relied on patient self-report, which may introduce information bias. Future research should integrate wearable devices (e.g., smart wristbands, Bluetooth-enabled weighing scales) or automated home weight data upload systems to enable objective remote monitoring of residential data, thereby improving data reliability and minimizing recall and reporting bias.

Based on the findings and limitations of this study, future research efforts could focus on the following directions to generate higher-level evidence and expand the application scope. First, Precision intervention: further investigate differentiated intervention protocols based on antipsychotic types, disease subtypes and genetic markers to achieve more targeted management (8). Second, digital follow-up: utilize wearable devices, mobile applications, or automated home weight data upload systems to enable automatic collection of home-based data and objective remote monitoring, thereby addressing the challenges of long-term adherence management (21, 22). Third, systematic ecologicalization: advocate the integration of core indicators for psychiatric weight management into hospital grading evaluations and specialized quality control systems, and explore medical insurance payment mechanisms tailored to its characteristics. This will further facilitate the ecosystem’s evolution from in-hospital intervention to whole-course management and policy support.

Although the 5–4-3 Model is feasible and effective in the short term, resource arrangement should be fully considered for its long-term application in general psychiatric settings. The upfront costs brought by a full-time multidisciplinary team and digital infrastructure may be compensated by fewer metabolic complications and readmissions. Future research should perform cost-effectiveness evaluation and examine how staff turnover affects the consistency of intervention delivery. As preliminary observations show, incorporating weight management metrics into current performance assessment and utilizing existing low-cost digital tools like WeChat helps maintain stable operation with limited extra funding.

## 5 Conclusion

The study results suggested that overweight and obese psychiatric inpatients achieved a significant initial weight loss in association with the 5–4-3 Model intervention. These patients sustained this weight loss effect during the 3-month follow-up after discharge. In a single-arm pre-post implementation, this systematic weight management intervention programme developed by our medical institution appears feasible and was associated with modest short-term improvements for overweight and obese individuals with mental disorders. However, causal attribution is limited by the uncontrolled design, and these findings should be considered preliminary.

## Data Availability

The raw data supporting the conclusions of this article will be made available by the authors, without undue reservation.
